# Reconstructing contact and a potential interbreeding geographical zone between Neanderthals and anatomically modern humans

**DOI:** 10.1038/s41598-024-70206-y

**Published:** 2024-09-03

**Authors:** Saman H. Guran, Masoud Yousefi, Anooshe Kafash, Elham Ghasidian

**Affiliations:** 1 Institute for Prehistoric Archaeology, University of Cologne, Cologne, Germany; 2 DiyarMehr Institute for Palaeolithic Research, Kermanshah, Iran; 3 Stiftung Neanderthal Museum, Mettmann, Germany; 4 Department of Biology, Hakim Sabzevari University, Sabzevar, Iran; 5 School of Culture and Society, Department of Archaeology and Heritage Studies, Aarhus University, Aarhus, Denmark

**Keywords:** Zagros Mountains, Ecological niche, Palaeoenvironment, Neanderthals, Anatomically modern humans, Climate sciences, Ecology, Environmental sciences

## Abstract

While the interbreeding of *Homo neanderthalensis* (hereafter Neanderthal) and *Anatomically modern human (AMH)* has been proven, owing to the shortage of fossils and absence of appropriate DNA, the timing and geography of their interbreeding are not clearly known. In this study, we applied ecological niche modelling (maximum entropy approach) and GIS to reconstruct the palaeodistribution of Neanderthals and AMHs in Southwest Asia and Southeast Europe and identify their contact and potential interbreeding zone during marine isotope stage 5 (MIS 5), when the second wave of interbreeding occurred. We used climatic variables characterizing the environmental conditions of MIS 5 ca. 120 to 80 kyr (averaged value) along with the topography and coordinates of Neanderthal and modern human archaeological sites to characterize the palaeodistribution of each species. Overlapping the models revealed that the Zagros Mountains were a contact and potential interbreeding zone for the two human species. We believe that the Zagros Mountains acted as a corridor connecting the Palearctic/Afrotropical realms, facilitating northwards dispersal of AMHs and southwards dispersal of Neanderthals during MIS 5. Our analyses are comparable with archaeological and genetic evidence collected during recent decades.

## Introduction

Following the ground-breaking discovery of biocultural admixture in the Late Pleistocene of different early human groups of Neanderthals, archaic/modern humans, and Denisovans, a large and growing body of research concerning the nature and evolutionary history of these events is presented. In addition to the significant consequences that the biological exchanges have had on species e.g.^1^ and related issues, the time^2–4^, and geography of contact and interbreeding are the subject of intense debate^5^. Neanderthals are an extinct lineage of hominins that emerged at approximately 400 kyr and died off at approximately 40 kyr.^6^ Fossil localities and morphological evidence of Neanderthals indicate that they are companionable with the Palearctic biogeographical realm, which includes from western Europe to the Altai Mountains in Siberia at 55° latitude and down to approximately 31° in Western Asia^7–10^. The chronological settlement patterns of the Neanderthals' sites indicate their expansion to the east and southwest Asia from at least 150 kyr^11^.

On the other hand, Anatomically Modern Humans (AMHs) have evolved in Africa for more than 300 kyr^12–14^. The evidence, including physical remains and morphological analyses, suggests that they exited Africa over and over during a period of at least 200 kyr^15–17^. AMHs also reached Eastern Asia at approximately 120 kyr^18^ and later reached Europe at approximately 60 kyr^19,20^. Recent accurate archaeological and palaeoenvironmental data suggest that AMHs rapidly adapted to the new and extreme environments beyond Africa, such as high plateaus, mountain systems and palearctic ecosystems^21^. Moreover, archaeological and fossil evidence indicates that AMHs entered southwestern Asia during MIS 5^15,22–24^.

There is strong evidence of multiple interbreeding events between two groups of Neanderthals and archaic/modern humans in western Eurasia e.g. ^3,25^. Moreover, many attempts have been made to estimate the timing of this interbreeding, and significant success has been achieved e.g.^26–28^ . Palaeogenetic studies have shown that the second wave of interbreeding occurred during MIS 5^4,28^ . In some studies, researchers have suggested that the lower latitude regions of southwestern Asia have high potential for the first overlap between Neanderthals and AMHs. Sanchez Goñi^29^ examined the patterns of expansion of Neanderthals and AMHs and showed that they shared the same ecological niches under certain climatic conditions during the Late Pleistocene. Recently, Churchill et al.^30^ reported facial morphological similarities between Neanderthals and AMHs in the Near East, indicating it could be a key region for interbreeding between the two lineages. However, it is still unclear where the two species met and interbred.

Ecological niche models (ENMs) are very practical tools for investigating the geography of two species’ palaeodistribution and potential interbreeding areas^31^. ENMs have been found to have important applications in palaeobiogeography, archaeology and palaeoanthropology^32–36^. They use occurrence data of a target species, including ancient humans, as well as palaeoenvironmental variables to calculate the probability of a species or hominin species’ presence in a defined geographic region^37^. These models have successfully been used to reconstruct the distribution of different hominin species^35,36,38^, identify refugia during the ice ages and reconstruct dispersal corridors^39^ , niche overlap among species^31^ and niche overlap with prey species^40^ . For example, Ruan et al.^31^ successfully used ENMs to identify the contact zones of Neanderthals and Denisovans. In another study, Benito et al.^36^ applied the ecological niches to determine the distribution of Neanderthals during the last interglacial period in Europe and in the Irano-Turonian region. Thus, ENMs can be used to model the palaeodistribution of Neanderthals and AMHs and locate the geography of their niche overlap^31,36^.

The aim of the present study was to reconstruct the palaeodistribution of Neanderthals and AMHs during MIS 5 to identify the contact and potential interbreeding geographical zones of these two species. We also estimated the most important predictor of the two species and investigated the responses of the two species to environmental variables. Previous studies have suggested Southwest Asia as a potential area for the interbreeding of Neanderthals and AMHs^30,41,42^ . Notably, this region which is located at the crossroads of the Afrotropical and Palearctic realms^43^ , matches the distribution of AMHs and Neanderthals, respectively. Thus, we hypothesized that these two species first met and interbred at the border of these two biogeographic realms where environmental conditions facilitated niche overlap and resource partitioning by providing a highly diverse habitat rich in resources. Climate is a major determinant of species distributions ^44^, particularly at large spatial scales, thus we expect climate to be more effective than topography in shaping the interactions between Neanderthals and AMHs.

## Results

### Reconstructing the contact and interbreeding zone

**Figure 1:**
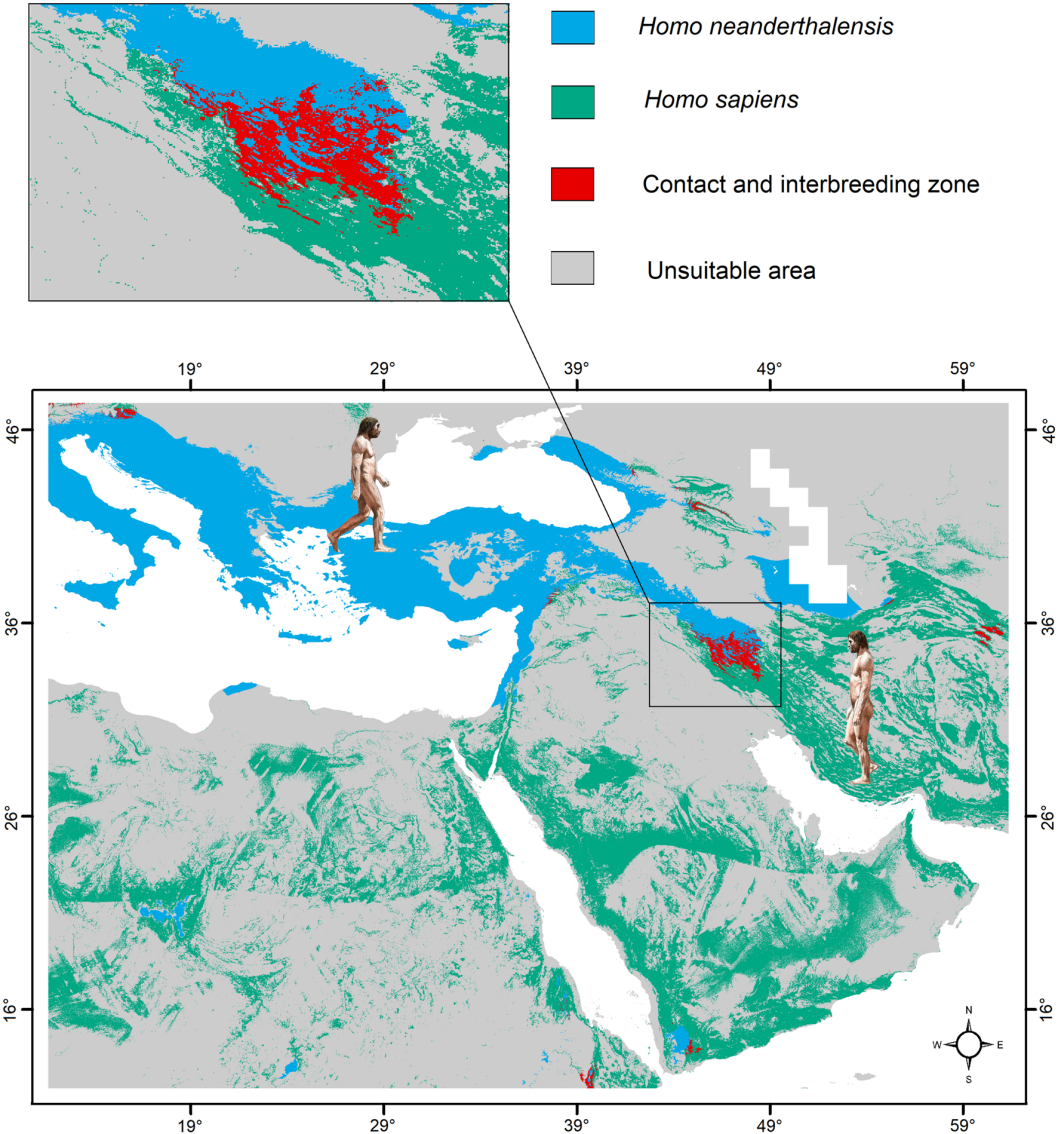
Habitat suitability models of the two *Homo* species and their potential contact and interbreeding zones in Southwest Asia and Southeast Europe. This figure was generated in QGIS 3.14.1 (www.qgis.org). The figures of the Neanderthal (left) and modern human (right) are adapted from www.demorgen.be.

The models developed in this study for Neanderthals (AUC = 0.941) and AMHs (AUC = 0.895) performed well according to the AUC model performance metric. Our model of the palaeodistribution of Neanderthals shows that north and west of the Mediterranean Sea towards the Levant, vast patches in Turkey, around the Black Sea, south of the Caspian Sea, Taurus, Caucasus and Zagros Mountains, were highly suitable for this species during MIS 5 (Fig. 1). The AMH palaeodistribution model identified large and continuous suitable patches in Africa, Arabia and the Iranian Plateau. Our model identified the Zagros Mountains as a contact and potential interbreeding zone in Southwest Asia and Southeast Europe.

### Variable importance and response curve

**Figure 2:**
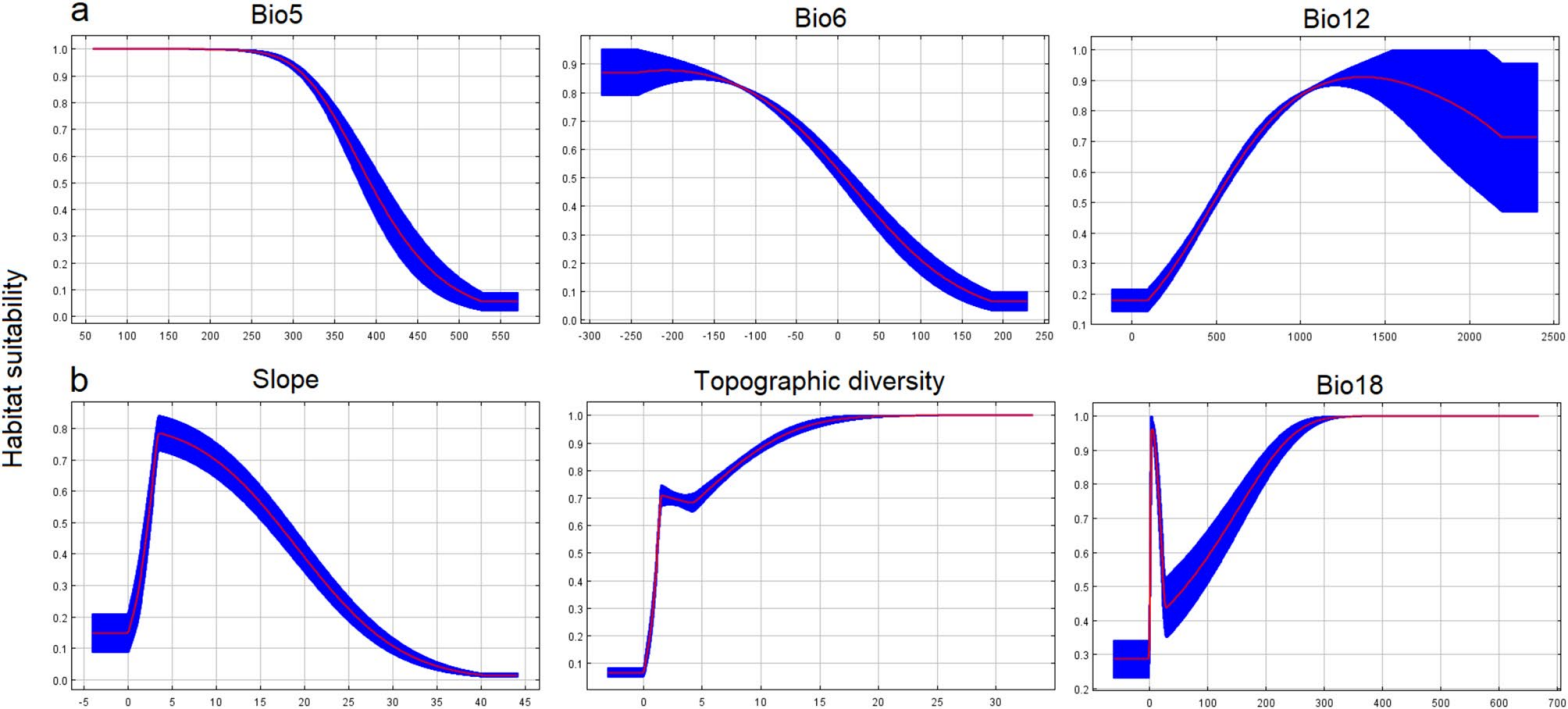
Response curves showing how the presence of Neanderthals (**a**) and AMHs (**b**) is related to the environmental variables (Bio5: maximum temperature of the warmest month, Bio6: minimum temperature of the coldest month, Bio12: annual precipitation and Bio18: precipitation of the warmest quarter) (https://biodiversityinformatics.amnh.org/open_source/maxent/).

We estimated the relative contributions of the environmental variables to the Maxent model of Neanderthals and AMHs. We found that the maximum temperature of the warmest month (with 58.5% contribution), the minimum temperature of the coldest month (with 19.7% contribution), and the annual precipitation (16.5% contribution) were the most important predictors of the palaeodistribution of Neanderthals^36^. The maximum temperature of the warmest month had a negative association with the presence of Neanderthals. Slope (with 35.6% contribution), topographic diversity (with 26% contribution) and precipitation of the warmest quarter (with 14% contribution) were the most important variables in shaping the palaeodistribution of AMHs. Both species presented similar responses to decreases in slope and habitat suitability in areas with high slopes. Figure 2 shows how each environmental variable affects the Maxent prediction for Neanderthals (a) and AMHs (b). The curves show how the predicted probability of presence changes as each environmental variable is varied, keeping all other environmental variables at their average sample value.

### Precipitation changes from 140 to 40 kyr

**Figure 3:**
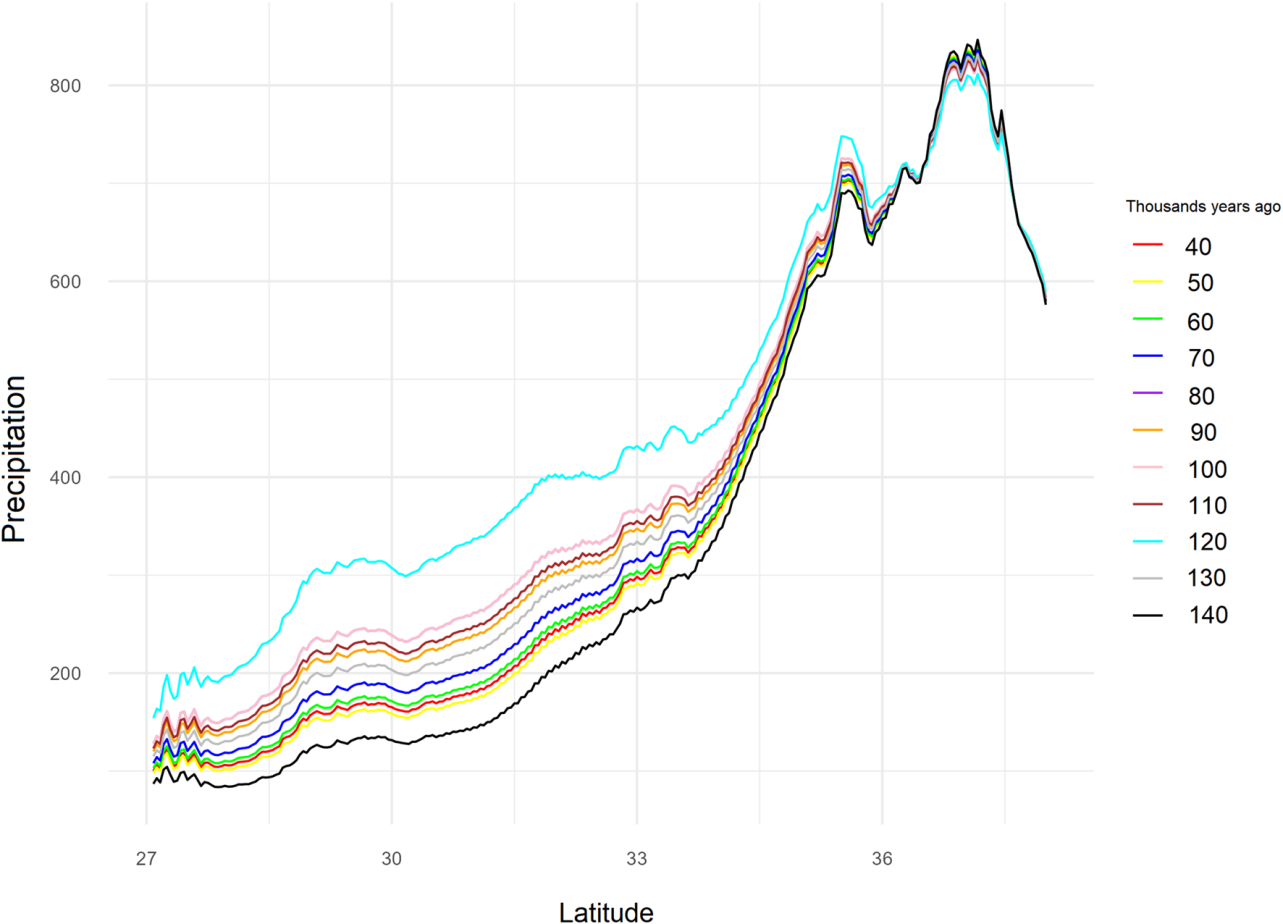
Precipitation changes from 140 to 40 kyr at 10,000 intervals.

Figure 3 shows the changes in precipitation from 140 to 40 kyr, with 10,000 intervals for the Zagros Mountains. The highest amount of precipitation occurred at 120 kyr, making it a suitable time for range expansion and interactions between Neanderthals and AMHs.

## Discussion

Hominin interbreeding is an important topic in palaeoanthropological studies, but when and where it occurred remain largely unknown. Among the different hominin species, the interbreeding of Neanderthals and AMHs is particularly important, as it contributes to the genetics of our own species. Here, we applied ENMs and GIS and revealed that the Zagros Mountains of Iran is a potentially highly suitable geographic unit for niche overlap and a potential interbreeding zone of these two species.

Our niche models predicted niche overlap for the two species in the Zagros Mountains. In support of this finding, various studies of genetic data ^e.g.^ ^45,46^, ecological modelling e.g.^21^ , archaeological and genetic records e.g.^24,46^ and fossils^30,47^ are in concordance with our niche overlap model. The expansion of Neanderthals to Zagros must have occurred in accordance with the Palearctic environment and karstic terrains from both sides of the Black Sea, i.e., the Caucasus and Anatolia crossing towards the southern regions. The latest evidence has shown that the southernmost expanse of Neanderthals was up to the latitudes of approximately 31° in an arm-shaped area that stretched to the south in two different directions alongside the Anti-Lebanon and Zagros Mountains^9^. The Neanderthals in territories further east, such as those found in present-day Uzbekistan^47^ , Tajikistan^48^ and Asian Russia^26,49^ , are known as Central and North Asian Neanderthals. To date, evidence of the presence of Neanderthals is consistent with southwestern humid mountainous zones, including Anti-Lebanon in the Levant^50^ , Anatolia^51^, the Caucasus^52,53^ and Zagros^9,54^.

The data on the MP period in the Zagros Mountains region are rich and more up-to-date due to the discoveries of stratified sites associated with absolute dates, hominin fossil records, and lithic artefacts. Among the large number of MP sites, four yielded Neanderthal fossils. The best-known of these is Shanidar Cave, where the remains of ten Neanderthals were discovered^55^. Approximately 350 km southeast (approximately 34° latitude), the Wezmeh and Bisetun caves in the Kermanshah region also yielded Neanderthal remains^9,54,56^. However, the recent discovery of Neanderthal remains from the Bawa Yawan Rock Shelter is significant since it yielded an in situ Neanderthal tooth in association with the Zagros Mousterian lithic artefacts^9^ . The tooth has been dated to around 65 kyr, whereas the age of the Mousterian layer, dates back to 83 kyr^57^.

Owing to the evidence of various hominin fossil remains, it has been determined that the region of Southwest Asia was inhabited by AMH modern humans in the Late Pleistocene. AMHs have inhabited the Levant during at least two periods between 177 and 194 kyr, as evidenced at the site of Misliya^16^ , and between ~ 120 kyr and 90 kyr, as shown at the sites of Skhul and Qafzeh^58^, before the area was permanently occupied by *H. sapiens* approximately 55,000 years ago^59^. There is a vast amount of data on hominin (including AMHs) occupations from 400 to 50 kyr in Arabia associated with Eastern African lithic technology see ^42^ and references therein, and moreover, physical remains, including AMH finger bones from Al-Wusta dated to ca. 85 kyr^15^, all indicate that Arabia was a gateway to Eurasia during the middle to late Pleistocene. There is evidence of the presence of nun-Mousterian MP artefacts dating back to 80 kyr in the southern regions of the Persian Plateau, both in the Zagros^60^ and in the southern to central parts of the Persian Plateau^24^.

In accordance with our initial expectation, the interaction and potential interbreeding zone of Neanderthals and AMHs was located in the contact zone of the Afrotropical and Palearctic realms, namely, the Zagros Mountains. There are several reasons why the Zagros Mountains are a suitable location for the niche overlap and potential interbreeding zone of two species. First, the Zagros Mountains are characterized by the environmental conditions of the Palearctic realm, which is the birthplace of Neanderthals^61^. At the same time, the areas surrounding Zagros are characterized by the environmental conditions of the Afrotropical realm, which is the birthplace of AMHs. Thus, the Zagros Mountains could have been visited repeatedly by people living in the border areas of the Palaearctic and Afrotropical realms during Pleistocene climatic shifts. Therefore, the possibility of interactions between different hominins, including AMHs and Neanderthals, was greater in these areas. Second, Zagros covers a vast geographical region (over 1500 km from Lake Van in Turkish Kurdistan to southeastern Iran) capable of supporting large stable human populations. Third, Zagros is exceptionally diverse in terms of topography and biodiversity^40,62–66^, making it capable of supporting the presence of two species at the same time. These mountains facilitate the niche overlap of some animal species with similar niches within the same habitat^40,67^. These mountains are known to play a very significant role in species distribution by acting as a dispersal barrier or as a dispersal corridor^63,65^. These findings support the results of our study.

Our findings are further supported by new fossil discoveries in the Zagros Mountains and new genetic data^46^. We assume a migration route into the Central Plateau from other directions, including the southern region via Arabia, the Persian Gulf, and the Oman Sea, is plausible^46^ . This route might have followed the coastal lines towards the north and eventually into the inner parts of the Persian Plateau. Recent evidence of hominin occupations scattered on the surface in areas located in the southernmost part of the Persian Plateau supports our hypothesis^24,60,68^.

Our initial supposition was that climatic factors would be the predominant force in predicting the distributions of both Neanderthals and AMHs. However, our findings revealed a nuanced picture: while climate emerged as the key determinant of the Neanderthal habitat, AMH distribution was significantly influenced by topographical variations. The climate was homogenous, but the topography was heterogeneous across the AMH distribution areas. These findings likely suggest that topography played a more pronounced role in sculpting the distribution pattern of AMHs. Our study contributes to the growing body of evidence that underscores the complex interplay between environmental factors in determining species distributions. Our results are in line with prey overlap^40^, showing that the annual precipitation and maximum temperature of the warmest month were the most important predictors of Neanderthal distribution on the Persian Plateau. Climate was the most important determinant of Neanderthal distribution in Europe and the Iran–Turanian region during the last interglacial period; however, the influence of topography was confined to local scales^36^.

One particular application of ecological niche models (ENMs) is to identify suitable areas for the presence of target species where no observations have been made^69,70^. Field surveys guided by ENMs have led to the discovery of new populations and rare species^69,70^, thereby proving the utility of ENMs in this context. Our model, which predicts the interbreeding areas of Neanderthals and AMHs, is assigned a very high priority for future field investigations and excavations. Although field testing of ENMs in archaeological studies is limited^40^, we encourage Iranian archaeologists to conduct field excavations in this potential interbreeding area to evaluate the practicality of the models in archaeological research. Moreover, the use of ENMs can guide the allocation of resources for archaeological excavations, ensuring that efforts are concentrated in areas with the highest potential for significant findings. By prioritizing these high-probability locations, researchers can maximize the efficiency of their fieldwork, leading to more targeted and fruitful excavations.

## Conclusions

Before this study, our understanding of the interbreeding of AMHs and Neanderthals was based on genetic and morphology data alone^71,72^. For the first time, we applied ENMs as additional and independent lines of information to locate possible geographic locations where the two species interbred. Our study identified the Persian Plateau, particularly the Zagros Mountains, as a potential interbreeding area for AMHs and Neanderthals. The possibility of attracting different hominin groups in the Zagros Mountains is justified by the geographical conditions of this region, since it is located in two different biogeographical zones, namely, the Palearctic and Afrotropical realms. The border areas of two realms are important in biology since they operate as refugia for species from glacial environments. Consequently, some parts of the Zagros Mountains could have been visited repeatedly by people living in the border areas of the Palaearctic and Afrotropical realms during Pleistocene climatic shifts. Therefore, the possibility of interaction between different hominins, including AMHs and Neanderthals, was greater in these areas.

In addition to our findings that the Persian Plateau served as a hub for *Homo sapiens* after dispersal from Africa^46^, we conclude that this plateau contributed significantly to hominin distribution^40,62,73^ , dispersal^24,74,75^ and evolution^46,76^, and we await many exciting discoveries that will shed light on human evolution and dispersal.

## Methods

### Archaeological sites

**Figure 4:**
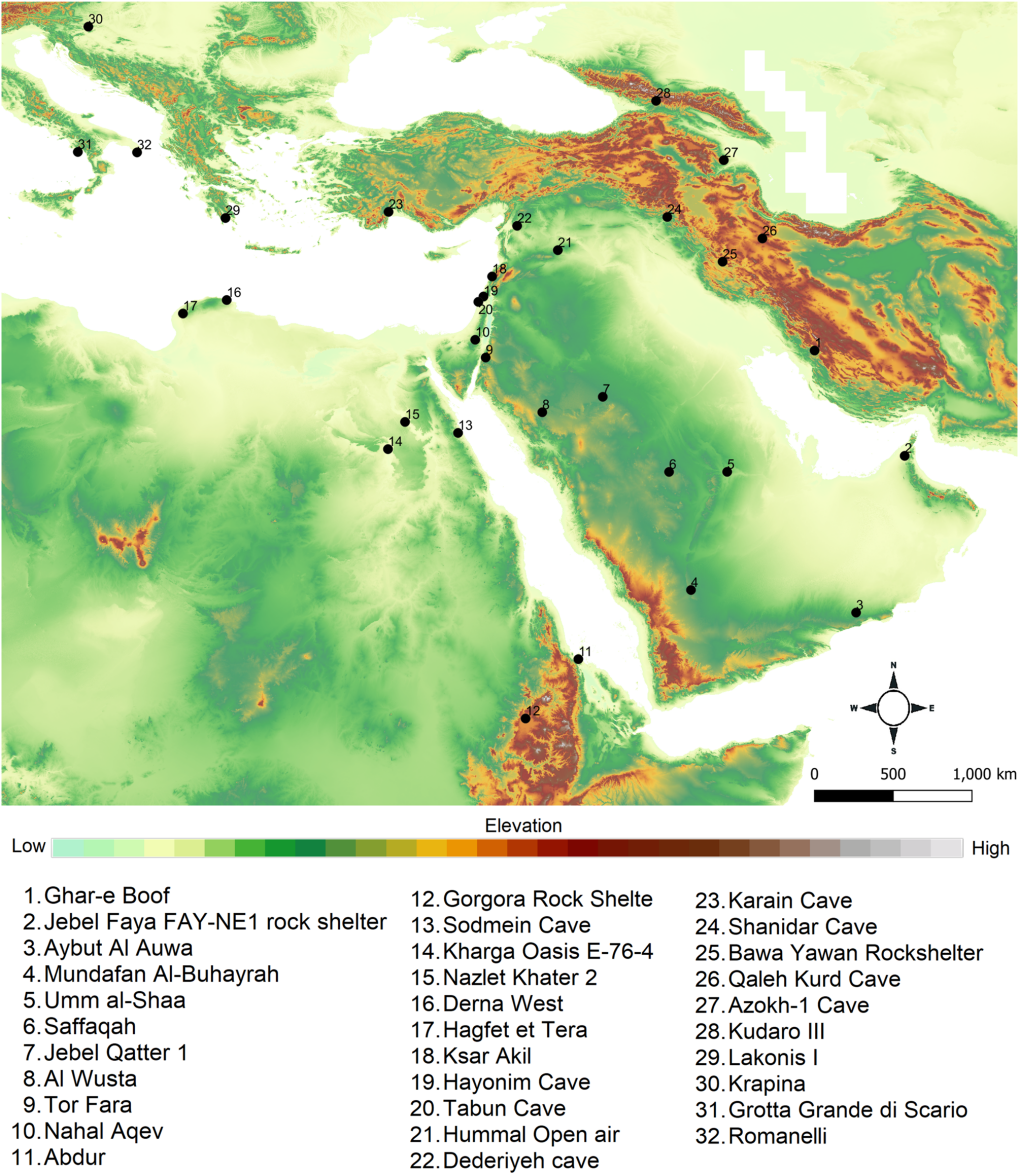
Topographic view of the study area showing the distribution of key archaeological sites attributed to Neanderthals and/or humans dating between MIS 5 and 3. These well-known archaeological sites are presented here to provide an overview of a potential interbreeding zone and are not used exclusively for niche modeling. Map data acquired from http://www.roceeh.org and created in www.qgis.org.

We obtained 38 occurrence points for Neanderthals and 45 for AMHs (Fig. 4), extracted from multiple sources, including the “Role of Culture in Early Expansions of Humans Out of Africa” (ROCEEH: http://www.roceeh.net) Database (ROAD30,31) and Appendix S1 in Benito et al.^36^. Each archaeological site was associated with one or two species on the basis of fossil records and lithic artefacts. Since our research focuses on the time frame MIS 5 (e.g., 120–80 kyr), we used only the archaeological sites during this period for southwest Asia and southeast Europe. We carefully examined each coordinate and removed the duplicates. Since the environmental data were at a spatial resolution of ~ 5 km (4.65 km at the equator), we thinned the occurrence data to 5 km to avoid pseudoreplication^40^. This time frame was selected because it is suggested that interbreeding events take place during three different periods^28^. The initial wave of interbreeding occurred ~ 250 to 200 kyr, the second wave of interbreeding occurred ~ 100 to 120 kyr and the third and last interbreeding occurred ~ 60 to 50 kyr. We were unable to find enough archaeological sites associated with the presence of the two species for the first interbreeding event to construct robust niche models; thus, we focused on the second interbreeding event that occurred during MIS 5^28^.

### Environmental predictors

We considered environmental variables related to past climate and topography to reconstruct the AMH and Neanderthal niches during MIS 5. As palaeoclimatic variables, we added the maximum temperature of the warmest month, the minimum temperature of the coldest month, the annual precipitation and precipitation of the warmest quarter to the niche models for the MIS 5-time span. Palaeoclimatic data were obtained from Oscillayers, which is a dataset of climatic oscillations over Plio–Pleistocene time scales at high spatial–temporal resolution^77^. We estimated the average values for each of the abovementioned variables during MIS 5 via the raster package v. 3.4–13^78^ implemented in the R environment^79^. To consider topography, we included the slope and topographic heterogeneity^36,40^, which were downloaded from EarthEnv^80^. To avoid multicollinearity among the predictors, we calculated a variance inflation factor (VIF;^81^) via the “vifstep” function in the “usdm” package^82^ and ensured that the collinearity among the predictors was low (VIF < 10).

### Ecological niche modelling

In this study, we used the maximum entropy modelling approach^83^ to reconstruct ecological niche models of Neanderthals and AMHs during MIS 5. Maxent version 3.4.4 with default settings was used to build the niche models^84^. We used the Maxent model because it has been shown to perform better than other niche modelling methods^37,84^. Maxent was run with maximum iterations of 500, convergence threshold of 0.0001, and 5000 randomly selected background points across the study area. We then overlapped the two palaeodistribution models to identify potential areas for their contact zones in the QGIS (www.qgis.org). The performance of the niche models was assessed via the area under the curve (AUC) metric of the receiving operator characteristic (ROC) curve^83^. An AUC value of 0.5 indicates that the performance of the model is not better than random, whereas values closer to 1.0 indicate better model performance^85^. The ROC curves were created by selecting 80% of the data for training and 20% for testing.

## Data Availability

All data needed to evaluate the conclusions in the paper are present in the paper or the references cited here within. We obtained archaeological sites data from the ROCEEH Out of Africa Database (ROAD) (http://www.roceeh.org) and references cited in the manuscript.
