# Correction: Reconstructing contact and a potential interbreeding geographical zone between Neanderthals and anatomically modern humans

**DOI:** 10.1038/s41598-026-50286-8

**Published:** 2026-05-01

**Authors:** Saman H. Guran, Masoud Yousefi, Anooshe Kafash, Elham Ghasidian

**Affiliations:** 1 Institute for Prehistoric Archaeology, University of Cologne, Cologne, Germany; 2 DiyarMehr Institute for Palaeolithic Research, Kermanshah, Iran; 3 Stiftung Neanderthal Museum, Mettmann, Germany; 4 Department of Biology, Hakim Sabzevari University, Sabzevar, Iran; 5 School of Culture and Society, Department of Archaeology and Heritage Studies, Aarhus University, Aarhus, Denmark

Correction to: *Scientific Reports* 10.1038/s41598-024-70206-y, published online 03 September 2024

The original version of this Article contained errors.

In the Results section, under the subheading ‘Reconstructing the contact and interbreeding zone’, the reported AUC values were missing decimal points. As a result,

“The models developed in this study for Neanderthals (AUC = 941) and AMHs (AUC = 895) performed well according to the AUC model performance metric.”

now reads:

“The models developed in this study for Neanderthals (AUC = 0.941) and AMHs (AUC = 0.895) performed well according to the AUC model performance metric.”

Additionally, in the Methods section, under the subheading ‘Ecological niche modelling’, some methodological detail was omitted. As a result,

“In this study, we used the maximum entropy modelling approach^83^ to reconstruct ecological niche models of Neanderthals and AMHs during MIS 5. Maxent version 3.4.4 was used to build the niche models^84^. We used the Maxent model because it has been shown to perform better than other niche modelling methods^37,84^. We then overlapped the two palaeodistribution models to identify potential areas for their contact zones in the QGIS (www.qgis.org).”

now reads:

“In this study, we used the maximum entropy modelling approach^83^ to reconstruct ecological niche models of Neanderthals and AMHs during MIS 5. Maxent version 3.4.4 with default settings was used to build the niche models^84^. We used the Maxent model because it has been shown to perform better than other niche modelling methods^37,84^. Maxent was run with maximum iterations of 500, convergence threshold of 0.0001, and 5000 randomly selected background points across the study area. We then overlapped the two palaeodistribution models to identify potential areas for their contact zones in the QGIS (www.qgis.org).”

Finally, the legend of Figure 4 has been corrected.

“Distribution of key archaeological sites dating between MIS 5 to 3 across southwest Asia and southeast Europe. Map data acquired from http://www.roceeh.org and created in www.qgis.org.”

now reads:

“Topographic view of the study area showing the distribution of key archaeological sites attributed to Neanderthals and/or humans dating between MIS 5 and 3. These well-known archaeological sites are presented here to provide an overview of a potential interbreeding zone and are not used exclusively for niche modeling. Map data acquired from http://www.roceeh.org and created in www.qgis.org.”

The original Article has been corrected.

